# Artificial intelligence in electroencephalography analysis for epilepsy diagnosis and management

**DOI:** 10.3389/fneur.2025.1615120

**Published:** 2025-08-18

**Authors:** Chenxi Wang, Xinyue Yuan, Wei Jing

**Affiliations:** ^1^Shanxi Medical University, Taiyuan, Shanxi, China; ^2^Third Hospital of Shanxi Medical University, Shanxi Bethune Hospital, Shanxi Academy of Medical Sciences, Tongji Shanxi Hospital, Taiyuan, China

**Keywords:** epilepsy, electroencephalography, artificial intelligence, deep learning, machine learning, multimodal data fusion

## Abstract

**Introduction:**

Epilepsy is a prevalent chronic neurological disorder primarily diagnosed using electroencephalography (EEG). Traditional EEG interpretation relies on manual analysis, which suffers from high misdiagnosis rates and inefficiency.

**Methods:**

This review systematically evaluates the integration of artificial intelligence (AI), particularly deep learning (DL) and machine learning (ML), into EEG analysis for epilepsy management. We focus on two dominant AI-EEG application models: supportive AI (augmenting clinical decisions) and predictive AI (anticipating seizures or outcomes).

**Results:**

AI-based EEG analysis demonstrates significant potential in improving epilepsy detection, monitoring, and therapeutic evaluation. Key advancements include enhanced precision, efficiency, and capabilities for multimodal data fusion and personalized diagnosis. However, challenges persist, such as limited model interpretability, data quality constraints, and barriers to clinical translation. Crucially, AI outputs require clinician verification alongside multidimensional clinical data.

**Discussion:**

Future research must prioritize algorithm optimization, data quality improvement, and enhanced AI transparency. Interdisciplinary collaboration is essential to bridge the gap between technical innovation and clinical implementation. This review highlights both the transformative potential and current limitations of AI-EEG in epilepsy care, providing a roadmap for future developments.

## Graphical abstract

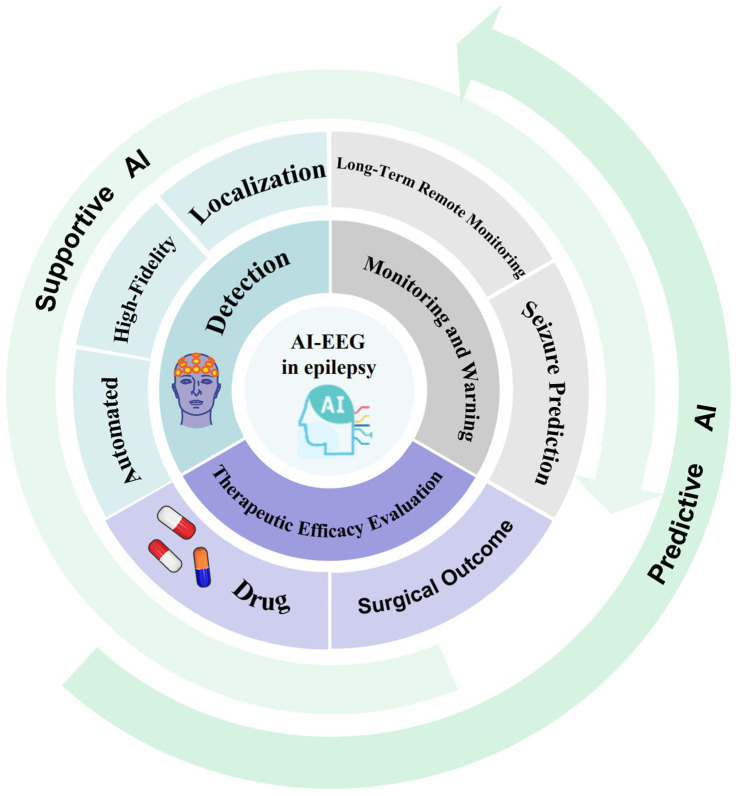

## 1 Introduction

Epilepsy affects approximately 50 million individuals globally, including 10 million patients in China (1, 2), underscoring its status as one of the most prevalent chronic neurological disorders worldwide. The World Health Organization (WHO) estimates that nearly 70% of patients with epilepsy can achieve freedom from seizures with appropriate diagnosis and treatment (3). However, owing to diagnostic and therapeutic gaps, approximately two-thirds of epilepsy patients in China fail to receive adequate treatment (1). Electroencephalography (EEG) is the most critical auxiliary diagnostic tool for epilepsy, and plays a critical role in the diagnosis, classification, and localization of epileptogenic foci. Theoretically, all epileptic seizures can be associated with detectable epileptiform discharges on EEG (4), but the clinical application of this diagnostic tool is subject to several limitations. First, conventional extracranial EEG has relatively low spatial resolution, covering only approximately one-third of the scalp, which limits its ability to detect deep-seated epileptogenic foci or abnormal activity in regions that are not covered (5). Second, technical inconsistencies, such as electrode placement errors or insufficient recording duration, may compromise data quality (6). Consequently, these factors yield detection rates of 40–50% for epileptiform discharges during initial routine EEG examinations. Even with 24-h prolonged EEG monitoring, the detection rate is only approximately 70–80% (4). Thus, an epilepsy diagnosis cannot be conclusively ruled out based on a normal EEG result (4). Furthermore, epileptiform discharges may also occur in other neurological conditions, such as autism spectrum disorder and Alzheimer’s disease (7, 8). Additionally, manual EEG interpretation is inherently subjective and inefficient, and the increasing volume of data exacerbates diagnostic variability (9). Traditional EEG systems also lack functionalities such as real-time analysis, closed-loop feedback, and predictive warnings. Therefore, achieving precise EEG data acquisition and efficient interpretation has become a critical research priority in recent years.

The rapid advancement of artificial intelligence (AI) technology offers innovative solutions for addressing these challenges and overcoming the limitations of EEGs (10). Artificial intelligence-based electroencephalography (AI-EEG), which uses technologies such as machine learning (ML), deep learning (DL), and multimodal fusion, enables high-quality data acquisition, real-time analysis, and automated seizure detection (11). This technology can significantly reduce the workload of EEG interpreters and improve diagnosis and treatment efficiency. In epilepsy management, AI applications can be categorized into two distinct paradigms. Supportive AI is designed to assist doctors in efficiently performing detection, recognition, and localization tasks. Predictive AI is centred on identifying characteristic or subtle patterns that are imperceptible to humans, with the aim of forecasting seizures and predicting treatment efficacy. Several studies have revealed that AI-EEG systems can achieve high-precision detection in specific tasks, such as the automatic identification of interictal epileptiform discharges. The performance of these methods is comparable to that of some individual experts and can even surpass that of other methods on standardized datasets (10). Concurrently, multimodal data integration and the application of wearable devices (12) have enabled a shift in epilepsy management from “postdiagnosis passive treatment” to “real-time prediction and proactive prevention,” which plays a crucial role in remote monitoring and predicting therapeutic outcomes. However, the development of AI-EEG faces challenges, such as limited model generalizability, data standardization issues, and ethical and privacy concerns. Importantly, despite the great potential of AI, contradictory findings must be considered, as well as the necessity of human supervision.

**Figure 1:**
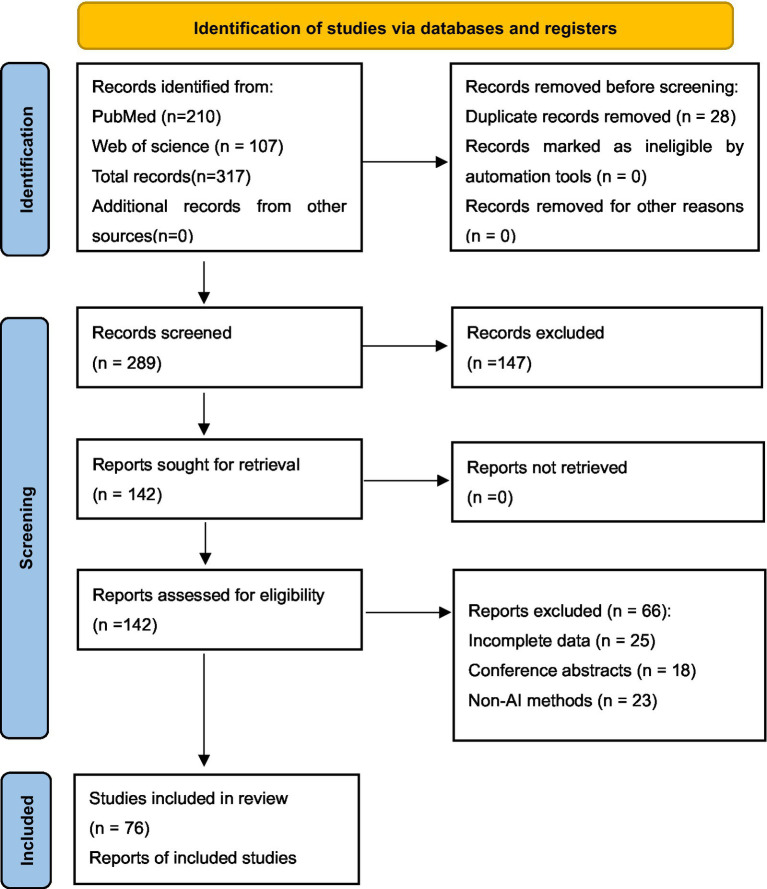
PRISMA flow diagram for systematic review of AI applications in epileptic EEG analysis.

In summary, numerous challenges remain in the diagnosis and treatment of epilepsy. Although EEG serves as a fundamental diagnostic tool with critical clinical importance, it also has inherent limitations. The continuous advancement of AI technologies has introduced new possibilities for epilepsy management through AI-EEG. Importantly, whether the technology in question is supportive AI or predictive AI, its core value lies in assisting rather than replacing human clinical decision-making. Despite demonstrating substantial potential, key challenges persist in overcoming technical and ethical barriers to promote its widespread clinical implementation. In this review, recent advancements in AI applications for EEG analysis are systematically examined; the current utilization of this technology in seizure detection, continuous monitoring, and therapeutic efficacy evaluation is explored; the capabilities and limitations of AI-EEG are critically assessed; and future research directions are proposed to address existing gaps in the field (Figure 1).

## 2 Literature search strategy

A comprehensive literature search was conducted in PubMed and Web of Science using the keywords “epilepsy,” “electroencephalography,” “EEG,” “artificial intelligence,” “AI,” “deep learning,” and “multimodal data fusion.” Priority was given to studies published in recent years, while those not directly relevant to the study topic or deviating from the core question were excluded. The focus remained on identifying high-quality, relevant research (Table 1).

**Table 1:** Technical specifications and performance metrics of intelligent epileptic EEG processing models.

Reference	Model name	Target application	Architecture	Input features	Sampling frequency	Electrodes (Count, position)	Training dataset	Data source	Segment duration	Validation method	Key advantages	Major limitations	Best performance
Tveit et al. (11)	SCORE-AI	Automated Interpretation of Clinical EEG	CNN	19-channel raw EEG + 1-channel ECG + Patient age and sex	256 Hz	19 scalp (10–20 system) + 1 ECG	30,493 records	Multicenter clinical EEG databases	Full recordings (mean 33 min)	Multicenter test (*n* = 100); Single-center test ( *n* = 9,785); Benchmarking ( *n* = 60)	Accessibility Enhancement; Clinical Safety Improvement; Expert-Level Diagnostic Accuracy; Workflow Optimization	Limited Patient Coverage; Dependency on Human Expert Labels	AUC:0.89–0.96 Specificity: 87.1% Accuracy: 88.3%
Chen et al. (13)	RF + CNN	Automated detection and classification of epileptic EEG states	CNN	Time-frequency; Nonlinear entropies	Bonn: 173.61 Hz New Delhi: 200 Hz	Bonn: Single-channel New Delhi: 10–20 system (count unspecified)	Bonn: 23.6 s per segment; New Delhi: 5.12 s per segment	Bonn EEG dataset; New Delhi EEG dataset	Bonn: 23.6 s per segment; New Delhi: 5.12 s per segment	Train-test split (3:1 ratio); No cross-validation mentioned	Unprecedented Accuracy; Effective Feature Fusion; Generalizability: High Computational Efficiency	Small Dataset; No Clinical Generalization; Unvalidated in Real-World Settings	Bonn Dataset:Accuracy: 99.2% Sensitivity: 99.42% Specificity: 98.82% Precision: 98.80% New Delhi Dataset: Accuracy: 100% Sensitivity: 100% Specificity: 100% Precision: 100%
Liu et al. (14)	Automatic Seizure Detection System	Epileptic seizure detection	S-transform + 15-layer CNN	S-transform spectrograms (35 × 32 × 6) from 1 s EEG segments	256 Hz	6 intracranial (grid/strip/depth)	5.5 h (21 seizures)	Freiburg iEEG	1 s segments	10-fold cross-validation	High temporal resolution	Limited to iEEG; Untested on scalp EEG; Patient-specific parameter tuning required	Segment-based: Sensitivity: 97.01% Specificity: 98.12% Event-based: Sensitivity: 95.45% FDR: 0.36/h
Li et al. (15)	FCNLSTM	Epileptic seizure detection	FC*N* + NLSTM	Raw EEG segments (1D time-series)	Bonn: 173.61 Hz Freiburg, CHB-MIT 256 Hz	Bonn Database: *Not specified* Freiburg Database Freiburg: 6 channels per patient Grid (g), Strip (s), Depth (d) CHB-MIT Database: 22 scalp electrodes	Bonn: 400 segments/class Freiburg: 564.03 h (87 seizures) CHB-MIT: 846.23 h (198 seizures)	Bonn, Freiburg, CHB-MIT databases	Freiburg, CHB-MIT:4 s Bonn:1024 points	Bonn:10-fold CV Freiburg/CHB-MIT: Patient-specific split	End-to-end; no manual feature engineering; captures temporal dependencies	Limited to patient-specific models; computational cost for nesting	Bonn: Accuracy: 98.44–100% Freiburg: Sensitivity: 97.47% FDR: 0.487/h CHB-MIT: Sensitivity: 95.42% FDR: 0.66/h
Shen et al. (16)	STFT+GoogleNet CNN	Real-time Seizure Detection	GoogleNet CNN (29 layers)	STFT spectrograms (120 × 344 matrix)	256 Hz	6 scalp channels: frontal/temporal/occipital	16 patients from CHB-MIT 10-min pre-seizure + 5-min post-seizure per patient	CHB-MIT	1.35-s sliding window	Leave-one-out cross-validation + 20% hold-out validation	Real-time processing (0.02 s/episode); low latency; high sensitivity	Cannot detect amplitude-depression seizures; limited to 2 Hz frequency resolution; GPU constraints exclude complex CNNs	Accuracy: 97.74% Sensitivity: 98.90% FPR: 1.94% Detection Delay: 9.85 s
Ansari et al. (19)	CNN-RF	Neonatal seizure detection	23-layer CNN; classifier: RF	Raw multi-channel EEG (0.5–15 Hz filtered)	256 Hz → 30 Hz	17, 13, or 9 electrodes (Fp1-2, F7-8, T3-4, T5-6, O1-2, F3-4, C3-4, P3-4, Cz)	26 neonates, 4,344 segments (50% seizure), augmented to >30,000 segments	NICU EEG recordings (Erasmus University Medical Center, 2003–2012)	90 s segments (60 s overlap for test)	Independent test set (22 neonates)	Automatic feature optimization; Shift-invariance; Faster recall than heuristic/feature-based; Retrainable	Training computationally heavy; Requires large dataset; Black-box interpretation; Performance drops for unseen seizure patterns	Full Test Set (22 neonates): Sensitivity: 77% False Alarm Rate: 0.9/h AUC: 83% After Excluding 7 Neonates: AUC: 88% False Alarm Rate: 0.73/h
Shama et al. (33)	DeepSOZ	Joint seizure detection and SOZ localization from scalp EEG	Transformer + LSTM + Attention-weighted Pooling	Multichannel EEG (1 s windows), positional embeddings	200 Hz	19 channels, 10–20 system	642 EEG recordings (10-min) from 120 adult patients	Temple University Hospital (TUH) corpus	1-s windows (full 10-min recordings)	Bootstrapped 5-fold nested CV	Robust multi-task performance, high-resolution predictions	Robust multi-task performance, high-resolution predictions	AU-ROC: 0.92 Sensitivity: 81% FPR: 0.44 min/h SOZ Accuracy: 74% (patient-level)
Shafiezadeh et al. (48)	CNN	Seizure forecasting	6-layer CN*N* + 2 dense layers (128/32 units), sigmoid output	Raw EEG signals	256 Hz → 128 Hz (downsampled)	CHB-MIT: 22 channels; Conegliano: 20 channels (10–20 system)	CHB-MIT (19 pts., 89 sz) + Conegliano (22 pts., 77 sz)	CHB-MIT, Conegliano (clinical EEG)	5 s windows	RCV, LOO, Cal1 (1 seizure), Cal2 (2 seizures)	Patient-independent calibration improves LOO performance by >20%; efficient fine-tuning (5–10 min)	Requires at least 1 seizure per patient for calibration	CHB-MIT: Accuracy: 69.35% Sensitivity: 69.74% Specificity: 69.90% Conegliano (Cal2): Accuracy: 70.67%Sensitivity: 75.37% Specificity: 71.28%

### 2.1 Database selection

A comprehensive literature search was conducted using the PubMed and Web of Science databases, known for their extensive coverage of medical, neuroscience, and engineering research. These databases were selected to ensure a thorough gathering of relevant studies on the intersection of AI and EEG in epilepsy.

### 2.2 Search time span

The search targeted literature published from 2012 to 2025, with a concentrated focus on the years 2020 to 2025. This timeframe was chosen due to the notable advancements and increased research activity surrounding AI technologies in the medical field, particularly in the application of EEG for epilepsy diagnosis and management.

### 2.3 Search keywords

A strategic combination of keywords was employed during the search process. The core keywords included:

- “Epilepsy”
- “Electroencephalogram”
- “Artificial intelligence”
- “Deep learning”
- “Multimodal data fusion”
- “Seizure prediction”
- “Treatment outcome”
- “Seizure detection”

To enhance the search breadth, specific machine learning algorithm names such as “convolutional neural networks,” “recurrent neural networks,” and “support vector machines” were also included. This approach aimed to capture diverse research addressing the application of various AI technologies in epilepsy EEG analysis.

### 2.4 Search strategy

Boolean operators (AND, OR, NOT) were utilized to create structured search expressions in each database. For instance, the search expression used in PubMed was:

(‘Artificial Intelligence’ OR ‘Machine Learning’ OR ‘Deep Learning’) AND (‘Electroencephalography’ OR ‘Epilepsy’ OR ‘Seizure Detection’) AND (‘EEG-based Epilepsy Care’)

This strategy was designed to optimize the precision and comprehensiveness of the search, allowing for effective identification of literature closely aligned with the review topic.

### 2.5 Literature screening process

#### 2.5.1 Inclusion criteria

The following criteria were established for the inclusion of studies:

- Research subjects must be epilepsy patients, utilizing EEG technology for monitoring and analysis.
- Studies must apply AI models or algorithms to process epilepsy EEG data, with objectives including seizure detection, focus localization, and disease prediction.
- Eligible literature types include original research, clinical trials, simulation studies, and retrospective analyses.
- Preference was given to studies featuring larger sample sizes, sound research designs, and credible findings.

#### 2.5.2 Exclusion criteria

Studies were excluded based on the following conditions:

- Absence of AI methods or models.
- Lack of relevance to epilepsy EEG diagnostics, monitoring, or prediction.
- Subjects not including epilepsy patients.
- Non-original research such as conference abstracts and reviews.
- Incomplete data or insufficient methodological detail.

### 2.6 Literature screening results

The implementation of the outlined search strategy resulted in the identification of 317 relevant articles. After an initial deduplication process, a preliminary screening was performed based on the established inclusion and exclusion criteria. This initial review involved examining titles and abstracts to filter out articles that did not meet the requirements. Following a thorough examination of the full texts of the remaining articles, a total of 76 articles were selected for inclusion in this review. These articles encompass a wide array of applications of various AI models in epilepsy EEG detection, monitoring, and therapeutic prediction, providing a robust data foundation for the review analysis.

## 3 Main text

### 3.1 Role of AI-EEG in epilepsy detection

The current research and application of AI-EEG technology in the field of epilepsy detection are primarily focused on the realm of supportive AI. This involves leveraging the robust computational and pattern recognition capabilities of AI to assist clinicians in more efficiently and accurately detecting, identifying, and localizing epileptic seizures, thereby providing a tool to increase diagnostic efficiency and detection standards.

#### 3.1.1 Automated detection

Traditional epileptic seizure detection typically relies on manual feature extraction. In contrast, AI-EEG leverages ML and DL models, such as convolutional neural networks (CNNs) and nested long short-term memory (NLSTM) models, to achieve automated seizure detection. Studies have demonstrated that a multi-feature extraction, fusion, and selection-based method for automated identification of epileptic EEG signals achieves exceptional accuracy across diverse datasets. This method first decomposes EEG signals using Discrete Wavelet Transform (DWT) and extracts hybrid features from sub-bands, including Approximate Entropy (ApEn), Fuzzy Entropy (FuzzyEn), Sample Entropy (SampEn), and Standard Deviation (STD). Secondly, the random forest (RF) algorithm is used for feature selection. Finally, a CNN is used to classify epilepsy EEG signals. In the single-channel scalp EEG dataset provided by the University of Bonn, the sampling frequency is 173.61 hertz. It includes five categories of signals: signals from healthy individuals with eyes open and closed, interictal signals from the contralateral and ipsilateral sides of the epileptic focus, and ictal signals. The model achieved a classification accuracy of 99.9% for interictal and ictal signals, with sensitivity and specificity reaching 100 and 99.8%, respectively. In the dataset collected at the New Delhi Neurology and Sleep Center, the international 10–20 system electrode layout is used with a sampling frequency of 200 hertz. It comprises three categories of signals: preictal, interictal, and ictal. The model demonstrated even more remarkable performance in classifying interictal and ictal signals, with accuracy, sensitivity, and specificity all reaching 100%. This method, which integrates time-domain and nonlinear entropy features and optimizes feature selection through RF, has significantly enhanced classification performance, thereby providing a high-precision solution for the automatic detection of epileptic seizures in clinical settings (13). Compared with the pure CNN-based method, a study based on the Stockwell transform (S-transform) and a deep CNN model can perform long-term intracranial EEG (iEEG) recordings and automatically detect epileptic seizures. The study employed S-transform and a 15-layer deep CNN to process 6-channel iEEG with a sampling rate of 256 Hz. Under high-resolution analysis based on 1-s segments, the system demonstrated excellent performance on 720 h of test data: sensitivity of 97.01% and specificity of 98.12% based on segment evaluation; sensitivity of 95% and a false positive rate(FPR) of only 0.36 per hour based on event evaluation. However, its application is currently limited to iEEG, requiring patient-specific parameter adjustments, and it has not been validated on scalp EEG (14).

In addition, an end-to-end automatic epileptic seizure detection system based on DL and utilizing NLSTM has been developed. This system can effectively explore the inherent temporal dependencies hidden in EEG signals by processing one-dimensional EEG data directly, without converting them into two-dimensional data. This approach not only alleviates computational pressure to some extent but also automatically extracts the intrinsic feature information of epileptic seizures without human intervention. The method initially employs a fully convolutional network with three convolutional blocks to learn representative epileptic seizure features from EEG data, with sampling rates of 173.61 Hz for the Bonn dataset and 256 Hz for the Freiburg/CHB-MIT dataset. Subsequently, these robust EEG features associated with epileptic seizures are presented as input to the NLSTM to explore the inherent temporal dependencies in EEG signals. Finally, the high-level features obtained from the NLSTM are fed into the softmax layer to output the predicted labels. The average sensitivity is 97.47%, specificity is 96.17%, and the false detection rate (FDR) is 0.487 per hour (15). The aforementioned studies demonstrate that AI-EEG, within the framework of Supportive AI, plays a significant role in substantially enhancing the automatic detection rate of epilepsy. This, in turn, greatly reduces the waste of human effort and time, achieving efficient detection of epilepsy.

The studies mentioned above indicate that within the framework of supportive AI, AI-EEG offers an efficient tool for the automatic detection of epileptic seizures. Its computational speed and high-precision performance in specific tasks hold promise for assisting physicians in reducing the human effort and time required to screen for epileptic seizure events in large amounts of EEG data. However, generalizing these models to broader and more complex clinical data still requires further validation, and the ultimate clinical confirmation will continue to rely on the professional judgement of physicians.

#### 3.1.2 High-fidelity detection

Epileptiform discharges in EEG exhibit distinct time-frequency characteristics and spatial distribution patterns. AI-EEG technology facilitates rapid and precise identification of epileptic seizures by employing multimodal signal processing techniques to extract these pathological signatures. For example, a real-time epilepsy detection system based on short-time Fourier transform (STFT) time-frequency analysis and a 29-layer GoogleNet CNN. The system directly processes 6-channel scalp EEG with a sampling rate of 256 Hz. It converts EEG into time-frequency spectrograms (120 × 344 matrices) via STFT as input, and utilizes the deep CNN to automatically extract frequency-domain features, thereby accurately detecting epileptic seizures. When evaluated on the CHB-MIT database, this method achieved an accuracy of 97.74%, sensitivity of 98.90%, and a FPR of 1.94% (16). The Standardized Computer-based Organized Reporting of EEG–AI (SCORE-AI) system demonstrates the capability to discriminate between abnormal and normal EEG recordings. The system utilizes 19-channel EEG + 1-channel ECG + demographic data, with an input sampling rate of 256 Hz. It was trained on a Nordic multicenter dataset comprising 30,493 records, with an average duration of 33 min. The area under the curve (AUC) on three independent test sets ranged from 0.89 to 0.96. The study showed that the specificity of SCORE-AI (90%) was significantly higher than that of the consensus of three human experts (73.3%) and individual experts (3–63%). Its sensitivity (86.7%) was comparable to that of human experts (93.3%), and the overall accuracy (88.3%) was also similar to that of human experts (83.3%). The system has been clinically validated and integrated into the Natus platform, but neonates and critically ill patients were excluded (11). This study provides a direct quantitative comparison between AI and human experts in a specific task, demonstrating AI’s advantage in specificity while its sensitivity is comparable but slightly lower than the expert consensus. This highlights the potential of AI as an auxiliary tool, especially in improving the consistency of interpretation. Recent studies have shown that a dynamic graph neural network (DNN) incorporating attention mechanisms can be effectively employed for precise epileptic seizure detection using single-channel EEG signals. Researchers conducted 12 classification tasks using the Bonn Epilepsy EEG Dataset, with experimental results showing optimal classification performance achieved through 25 runs of 10-fold cross-validation. The proposed model attained exceptional metrics including an accuracy of 99.83%, specificity of 99.91%, and sensitivity of 99.78% (17).

While the aforementioned methodologies are predominantly implemented using DL architectures, it should be noted that EEG signals inherently constitute nonlinear time-series data. Consequently, strategies relying solely on CNNs or DNNs may have inherent limitations in capturing the temporal characteristics of neural oscillatory activity. Furthermore, signals recorded from individual EEG channels inherently are composite measurements of attenuated cortical activity originating from multiple neuroanatomical regions. These signals are also contaminated by various physiological artifacts, including cardiac pulsations, electromyographic(EMG) interference, and ocular movement artifacts. To address these challenges, researchers must enhance the signal-to-noise ratio (SNR) and isolate the overlapping activities via spatial filtering—that is, linearly combining the EEG signals at multiple channels such that the sources of interest are enhanced and the unwanted sources are suppressed. Consequently, probabilistic-Common spatial patterns (P-CSP) have emerged as an advanced analytical framework for multi-channel EEG data interpretation (18). In addition, Ansari’s team developed an automated neonatal seizure detection system based on a hybrid model of a 23-layer CNN and a RF for the neonatal intensive care unit (NICU) setting. The model processes raw multi-channel EEG (filtered at 0.5–15 Hz, downsampled from 256 Hz to 30 Hz) via adaptive bipolar montage. It employs the CNN for automatic feature optimization and the RF classifier for decision-making. The study was trained on 4,344 segments from 26 neonates (50% seizure segments, augmented to >30,000 segments) and validated on an independent test set (22 neonates). The results demonstrated that the system is a reliable and accurate automated detection tool for the NICU, with test set performance achieving Sensitivity = 77%, False Alarm Rate (FAR) = 0.9/h, and AUC = 83% (AUC increased to 88% and FAR = 0.73/h after excluding specific cases). The study benefits from automatic feature optimization and translation invariance but faces challenges such as heavy computational burden, the need for large datasets, black-box interpretability, and generalizability. It is considered an experimental study. The research proves that a reliable and accurate automated neonatal seizure detector for continuous multi-channel EEG is an extremely useful support tool, especially for neonatal intensive care units (19). Meanwhile, an end-to-end DL model integrating CNN and Bidirectional LSTM (BLSTM) networks enables automated seizure detection in multi-channel EEG recordings. This model achieved an average seizure detection sensitivity of 0.91 across all patients (20). A novel hybrid 1D CNN-Bi LSTM model can be used for multi-channel EEG feature fusion to enable precise detection of epileptic seizures. The approach is validated using benchmark CHB-MIT dataset and 5-fold cross validation resulting in an average accuracy of 95.90%, with precision 94.78%, F1 score 95.95% (21).

Multimodal fusion technology integrating EEG analysis, motion analysis, and physiological variations such as heart rate, oxygen saturation, perspiration, and blood pressure fluctuations also has significant clinical value and substantial developmental potential for precise epileptic seizure detection (22). For example, a study evaluated a semi-automated multimodal wearable seizure detection framework using binaural temporal EEG (bte-EEG) and electrocardiogram (ECG). The bte-EEG of this framework employs two electrodes: a transcranial channel and a contralateral channel located in the hemisphere where the seizure originates. It uses a high-pass filter at 0.5 Hz and a low-pass filter at 35 Hz. The results showed that the average sensitivity was 59.1% when using only bte-EEG in visual analysis, but the average sensitivity increased to 62.2% after adding ECG, and the FAR decreased from 6.5 times per day to 2.4 times per day. However, the sensitivity of 62.2% and the preference for the temporal region suggest that lightweight devices still need to break through the limitations of spatial resolution (23).

Physiological artifacts including EMG, ocular movements, motion, ECG, pulse, and perspiration can interfere with epileptiform discharge detection, resulting in elevated FDR (24). Nevertheless, many novel deep learning frameworks are capable of maintaining high detection performance even in the presence of common EEG artifacts (25). Studies have shown that in the preprocessing of epilepsy EEG, independent component analysis (ICA) lays the foundation for subsequent precise analysis by separating the physiological artifact components from the EEG signals. The results indicated that the combination of ICA and Peak-to-Peak Amplitude Fluctuation (PPAF) achieved accuracy rates of 99, 98, and 95% for datasets with 3, 4, and 5 epileptic seizures, respectively. This further demonstrates the effectiveness of advanced preprocessing techniques (26). In addition, a study aimed at optimizing the separation of cardiac-related artifact components and for the first time achieved automatic joint removal of ECC and pulse interferences in neonatal EEG. This method is based on band-pass filtering, signal normalization, SOBI-ICA decomposition, and automatic component classification and reconstruction. The study used neonatal EEG data with 19 channels and a sampling rate of 256 Hz. It included 40 segments of 5-min clinical data, containing both seizure and non-seizure segments. By combining analysis with real ECG signals, the automatic artifact component identification achieved extremely high precision with an accuracy of 0.99, a false omission rate of 0.01, and a sensitivity of 0.93. It also effectively preserved brain activity, with an artifact power suppression index (API) reaching 98.22%, outperforming existing methods. This further demonstrates the important role of ICA in eliminating physiological artifacts. However, the model still has limitations, such as its dependence on synchronous ECG recording and lack of validation in cases with fewer than 19 channels. Further examination is needed for practical application (27). GCTNet, a Generative Adversarial Networks (GAN) guided parallel CNN and transformer network, enhances EEG artifact removal by preserving global consistency between denoised signals and clean reference data. This approach effectively eliminates physiological interference while ensuring signal fidelity, significantly improving the quality of subsequent analysis. Extensive experimental results have demonstrated that GCTNet significantly outperforms state-of-the-art networks in various artifact removal tasks. For example, in the task of removing EMG artifacts, GCTNet achieved an 11.15% reduction in Relative Root Mean Square Error (RRMSE) and a 9.81% improvement in Signal-to-Noise Ratio (SNR) over other methods (28). These studies reveal the effectiveness of AI methods in reducing artefact interference and enhancing EEG signal quality, which is crucial for subsequent precise identification. However, assessing the extent to which these artefact removal methods improve clinicians’ accuracy in identifying epileptic events requires more direct clinical research evidence.

At the same time, biomarkers also play an important role in the detection of epilepsy. Research has shown that Lempel-Ziv Complexity (LZC) in the alpha band may serve as a biomarker for diagnosing temporal lobe epilepsy (TLE) combined with cognitive impairment (CI). The study calculated 76 LZC features for 19 leads across four frequency bands (alpha, beta, delta, and theta). The study achieved a diagnostic efficacy of AUC 0.85 through an SVM model (29). In addition, studies have demonstrated that the alpha band PLVEEG and LZCEEG features can be further explored as potential biomarkers for anxiety disorders (AD) in patients with epilepsy (PWE). The K-Nearest-Neighbor (KNN) model has been shown to improve the AUC to 0.89 (30). At the same time, studies have constructed predictive models for identifying AD in PWE by combining clinical features with quantitative EEG (q EEG) features and using ML. The AUC of 0.96 effectively identified the presence of AD comorbidity in PWEs, complementing clinical diagnosis. This indicates that LZC features in the alpha band and Phase Locking Value (PLV) features in Fp2-O1 may be potential biomarkers for diagnosing AD in PWE (31).

#### 3.1.3 Preoperative localization

Accurate preoperative localization is important in epilepsy surgery, as it not only enables surgeons to delineate precise resection margins to avoid excessive tissue removal and minimize neurological deficits, but also prevents inadequate resection, thereby reducing the risk of postoperative recurrence. Conventional visual interpretation of EEG is limited by inherent limitations such as high interobserver variability and insufficient sensitivity, which may contribute to inaccuracies in localizing the epileptogenic zone. AI-EEG facilitates automated tracking of ictal activities, localization of seizure onset zones (SOZ), and construction of functional connectivity networks, thereby precisely identifying pathological brain regions and assisting clinicians in focusing on critical epileptogenic foci. For example, SZTrack is the first end-to-end seizure tracking network using scalp EEG. The study trains SZTrack using the cross-entropy loss between the seizure-level prediction P(Y^t = 1|X^t) and the clinicians’ annotation of whether a seizure is not occurring at time window t. The results demonstrate that SZTrack can automatically learn the spatial patterns of seizure propagation and is consistent with the observations of clinicians. It is a significant advancement in epilepsy localization technology (32). Concurrently, DeepSOZ has emerged as an alternative robust framework for seizure detection and SOZ localization through multichannel scalp EEG. The model is based on a Transformer encoder that fuses multi-channel temporal features and employs a bidirectional LSTM branch for seizure detection, combined with an attention-based multiple instance learning mechanism for SOZ localization. It utilizes clinical scalp EEG data with 19 channels and a sampling rate of 200 Hz, including 642 segments of 10-min recordings from 120 adult patients sourced from the TUH database, analyzed with a 1-s window. The model demonstrates excellent performance in seizure detection (AUROC = 0.94, sensitivity = 0.81) and achieves patient-level accuracy of 0.744 ± 0.058 and seizure-level accuracy of 0.731 ± 0.061 for SOZ localization, significantly outperforming traditional methods. Its strengths lie in the end-to-end joint task architecture and the ability to quantify uncertainty. However, it has limitations in distinguishing between anterior and posterior brain regions and has only been validated in cases of focal epilepsy (33).

High-frequency oscillations (HFOs) ranging from 80 to 500 Hz detected in EEG recordings have been identified as critical biomarkers of epileptogenic zones and can be utilized for preoperative localization of epileptogenic foci (34). However, the visual interpretation of HFO signals remains challenging owing to their sheer data volume, characterized by significant interrater variability, labor-intensive analysis processes, and heightened susceptibility to interpretive errors (35). To address this issue, researchers have developed an automatic HFO detection method based on a CNN. This method analyzes isolated peaks (“islands”) in time-frequency diagrams and can efficiently identify HFOs in long-term multi-channel iEEG data. The results showed that in the test of 7,940 samples, the precision was 94.19% and the recall was 89.37% (F1 = 91.71%), with high robustness against false HFOs caused by filtering artifacts (specificity reaching 94.19%). The detection of a single event took only 1–3 s, and the model training could be completed within 3 min (using a GTX 1080Ti GPU) (36). However, the invasive nature and substantial costs associated with iEEG-based localization present significant limitations on the clinical application of HFO detection in epilepsy management, highlighting the need for methodological refinements. Furthermore, recent advancements in noninvasive HFO localization techniques have shown promising outcomes. Emerging evidence indicates that scalp EEG and magnetoencephalography (MEG) can reliably capture HFOs in the 80–500 Hz range through non-invasive modalities (34, 37). Preliminary studies have already demonstrated the feasibility of combining noninvasive HFOs with DL. However, most of these methods are still in the laboratory stage and require large-scale clinical trials to verify their reliability and universality.

### 3.2 Role of AI-EEG in epilepsy monitoring and early warning

AI-EEG technology has two main application patterns in the field of epilepsy monitoring and early warning: supportive AI is used to achieve more efficient and convenient long-term monitoring and real-time seizure detection; predictive AI, on the other hand, is designed to utilize the powerful pattern recognition capabilities of AI to identify specific discharge patterns and subtle feature patterns in EEG signals that are difficult for humans to perceive, in order to predict future epileptic seizure events.

#### 3.2.1 Long-term remote monitoring

The progress of AI-EEG in the remote long-term continuous monitoring of epilepsy primarily reflects the value of supportive AI, which assists in achieving the broader and more convenient monitoring of epilepsy status through technological innovation. Conventional epilepsy monitoring has traditionally relied on in-hospital EEG recordings using standardized full-scalp electrode arrays combined with video surveillance, which has inherent limitations including challenges in implementing remote continuous monitoring, inability to perform real-time data analysis, and lack of closed-loop feedback mechanisms (38). AI-EEG can facilitate long-term remote monitoring of epilepsy through wearable EEG devices, DL models, and multimodal fusion technologies, supporting real-time or near-real-time seizure detection and promoting synchronized processing of targeted stimulation and data (39). Customized EEG wearable devices (waEEG) can enhance follow-up compliance compared to conventional ambulatory EEG (aEEG), providing a portable and effective solution for detecting interictal epileptiform activity in temporal lobe epilepsy (TLE) (40). Furthermore, an experimental system integrating a portable EEG recording device (ANT Neuro) with a non-invasive wrist-worn sensor (Fitbit Charge 3; Fitbit Inc.) and a smartphone application (Seer App; Seer Medical) has allowed epilepsy patients to autonomously, continuously, and safely acquire noninvasive variables during home-based monitoring (41). Results showed that remote training and support enabled the successful implementation of novel non-invasive technologies for independent home use. However, ensuring long-term acceptability and usability requires systematic integration into patients’ daily routines with healthcare provider involvement, coupled with sustained support delivery and personalized feedback mechanisms (42). These devices show enhanced wearability while effectively mitigating psychological distress associated with conventional monitoring systems, eliminating the need for cumbersome head-mounted apparatus and reducing social stigma and self-perceived stigmatization. Furthermore, the integration of AI with telemedicine platforms not only addresses the care gap for epilepsy patients in resource-limited regions but also holds significant developmental potential for advancing remote long-term monitoring paradigms in epileptology (43). AI-EEG enables direct communication pathways between the human brain and external environments through brain-computer interfaces (BCIs), enabling targeted neuromodulation and closed-loop feedback mechanisms (44). The findings showed that prolonged intracranial monitoring, when combined with responsive stimulation over months to years, is essential for understanding the dynamic nature of epilepsy circuits *in vivo* (45). However, current AI-based long-term remote EEG monitoring technologies still have many limitations and limited research results. The primary functions of these AI technologies remain focused on supporting real-time and near-real-time events, such as seizure detection and status monitoring, which are in the category of supportive AI. Further exploration in this area is still needed in the future.

The development of AI-EEG technologies is essentially aimed at providing clinicians with new supportive tools. Their core value lies in assisting doctors in more comprehensively assessing treatment responses through automated feature extraction, efficient data processing, and pattern recognition. These features contribute to optimizing individualized decision-making. Clearly, such technologies do not replace clinical judgement. The analysis results they generate must be verified and interpreted by doctors in conjunction with multidimensional information on the patient’s medical history, physical examination findings, and imaging results. The ultimate diagnostic and therapeutic decisions must be led by the doctor, with AI serving only as an auxiliary support system to enhance the doctor’s decision-making capabilities.

#### 3.2.2 Seizure prediction

Epileptic seizure prediction is a core application area of predictive AI in the field of epilepsy. Its core goal is to utilize AI algorithms, particularly DL, to analyze EEG signals and automatically identify and interpret subtle and complex feature patterns that are difficult for humans to recognize directly or understand. This enables early warnings to be communicated several minutes to several hours before an epileptic seizure occurs.

Seizure prediction represents a critical application of AI-EEG in clinical epilepsy monitoring. Conventional seizure prediction methods have limitations in accuracy, real-time performance, and generalizability. In contrast, AI-EEG leverages multimodal data fusion and DL techniques to automatically extract and analyze complex features within EEG signals, thereby improving warning sensitivity and specificity (46). A study has introduced LSTM networks into the interpretation of EEG, using temporal and frequency domain features between the inter-channel correlation of EEG and graph theory features to predict epileptic seizures. The results showed that this model achieved high sensitivity in predicting epileptic seizures and a low FPR of 0.11–0.02 times per hour. Compared with traditional ML techniques and convolutional CNNs, the LSTM-based method significantly improved the performance of epileptic seizure prediction (47). In addition, a study has proposed a CNN model based on EEG signal feature fusion and patient calibration, employing rigorous leave-one-patient-out validation. The model directly processes raw EEG signals, with a sampling rate downsampled from 256 Hz to 128 Hz, using 22 or 20 channels of scalp EEG corresponding to the CHB-MIT and Conegliano datasets, respectively. A 5-s analysis window is used to define preictal and interictal states. The study innovatively introduces a two-stage calibration mechanism, Cal1 and Cal2. The findings indicate that on the CHB-MIT dataset, the leave-one-patient-out validation baseline accuracy was 53.55%, which increased to 65.77% after single-seizure calibration (Cal1) and reached 69.35% after dual calibration (Cal2). Sensitivity increased from 40.28 to 62.75 and 69.74%, respectively. The Conegliano dataset showed a similar trend, with Cal2 increasing accuracy from 45.72 to 70.67%. However, the framework has limitations: calibration requires individual seizure records (≥1) and ≥4 h of preictal EEG, making it inapplicable to high-risk patients who have not had seizures. Performance gains also showed significant patient variability (ACC increased by 12–47%), and long-term stability was not verified. Despite these limitations, the study was the first to demonstrate that only 1 to 2 seizure events are needed for fine-tuning to significantly enhance cross-patient prediction performance, offering a new paradigm for clinical generalization. Future work needs to explore calibration strategies without seizures (48). Furthermore, the Long-term Recurrent Convolutional Network (LRCN) has shown superior performance in early seizure prediction. This model achieved 93.40% accuracy, 91.88% sensitivity, and 86.13% specificity in segment-based evaluation (49). A tiny one-dimensional stacked CNN (1D-SCNN) based on STFT also has shown efficacy in seizure prediction. This architecture was evaluated on the test set of the American Epilepsy Society Seizure Prediction Challenge dataset, this architecture achieved an average sensitivity of 94.44%, average FPR of 0.011/h and average AUC of 0.979 (50).

These studies reveal that predictive AI models hold great potential for providing early warnings of epileptic seizures. Their core advantage lies in their ability to process vast amounts of EEG data and identify and learn subtle or complex feature patterns related to impending epileptic seizures that are too faint or intricate for human experts to discern. This advancement not only represents a technological breakthrough but also lays the foundation for the future exploration of the pathophysiological mechanisms of epileptic seizures and individualized predictive treatment responses.

### 3.3 Role of AI-EEG in epilepsy therapeutic efficacy evaluation

AI-EEG also plays an important role in the prediction and evaluation of epilepsy treatment efficacy, covering multiple dimensions such as drug efficacy prediction and surgical prognosis assessment. Efficacy prediction represents a core application area of predictive AI in epilepsy management. Its goal is to use current data to prospectively predict patients’ future responses to specific treatments. Notably, accurate and efficient predictive modelling heavily depends on supportive AI technologies, including automated EEG feature extraction, signal preprocessing and artefact removal, as well as multimodal data fusion. These supportive AI technologies provide high-quality, information-rich input features for predictive models.

#### 3.3.1 Drug efficacy evaluation

Predictive AI models utilize key EEG features extracted by supportive AI technologies alongside clinical data to predict drug responses. AI-EEG can assist in the prospective assessment of patients’ responses to antiseizure medications (ASMs), providing a reference for optimizing individualized treatment plans. For instance, a personalized prediction model integrating EEG complexity and 15 clinical features has shown the ability to predict responses to oxcarbazepine (OXC) monotherapy in patients with focal epilepsy, thereby improving the precision of initial drug selection. The prediction models were gradient boosting decision tree-Kolmogorov complexity (GBDT-KC) and gradient boosting decision tree-Lempel-Ziv complexity (GBDT-LZC). Results showed that the GBDT-LZC model had an average accuracy of 81% and a sensitivity of 91%. The GBDT-KC model had an average accuracy of 82% and a sensitivity of 83% (51). Another study showed that 19-channel EEG can be used to predict the response of patients with temporal lobe epilepsy (TLE) to levetiracetam (LEV) through ML methods (52). Meanwhile, an experiment used quantitative EEG(QEEG) features from children with absence epilepsy and ML to predict the therapeutic effect of valproic acid in this population. The results showed that K-nearest neighbor (KNN) classification using theta-band power in the temporal lobe yielded the best performance, with a sensitivity of 92.31%, specificity of 76.92%, accuracy of 84.62%, and an AUC of 88.46%. The study demonstrated good sensitivity, but the specificity of 76.92% indicates that there is still a certain proportion of false-positive predictions, which may lead to unnecessary drug trials or concerns (53). Similarly, other studies have also pointed out that AI models may face the challenge of insufficient specificity in epilepsy-related predictions. For example, Brigo et al.’s study showed that ChatGPT performed well in diagnosing epilepsy syndromes (*κ* = 1.00) and structural etiologies (accuracy = 90.0%), but it performed poorly in ambiguous cases such as unknown seizure types (accuracy = 12.5%) and rare etiologies (54). Additionally, a study developed a ML model based on the XGBoost algorithm. Demographic characteristics, medical history, and features from auxiliary examinations such as EEG and magnetic resonance imaging (MRI) were selected to distinguish patients with different remission outcomes. This method accurately predicts the efficacy of ASMs in treating epilepsy patients, achieving optimal predictive performance for ASM treatment effects between patients with remission and those with no remission, with an F1 score of 0.947 and an AUC value of 0.979 (55). The development of these technologies helps provide each patient with more effective ASMs.

Although these results are encouraging, high AUC values do not always fully translate into high precision in clinical practice, and the generalizability of the models and their performance on unseen data still need to be validated in larger-scale, multicenter prospective studies. More importantly, as demonstrated by the aforementioned studies on AI models, even large language models such as ChatGPT may experience reduced specificity and misclassification issues when it comes to complex clinical judgments, such as epilepsy diagnosis and classification. This highlights the necessity of human oversight and clinical integration (54).

The development of these AI-EEG technologies has demonstrated the great potential of AI in optimizing epilepsy drug treatment decisions. Their core value lies in providing objective data-driven information to support decision-making for the individualized selection of more effective antiepileptic drugs. However, current AI models still face challenges in predicting drug treatment responses, such as the limited interpretability of specific models, high dependence on data quality and feature selection, and the observed lack of specificity and potential risk of misclassification in some studies (54).

The development of these AI-EEG technologies has demonstrated the great potential of AI in optimizing epilepsy drug treatment decisions. Their core value lies in providing objective data-driven information to support decision-making for the individualized selection of more effective antiepileptic drugs. However, current AI models still face challenges in predicting drug treatment responses, such as the limited interpretability of specific models, high dependence on data quality and feature selection, and the observed lack of specificity and potential risk of misclassification in some studies (54). Therefore, AI-generated predictive results should be regarded as a powerful auxiliary tool that enhances the information basis for clinical decision-making. The final clinical application requires clinicians to integrate the insights provided by AI, individual patient circumstances, clinical experience, and other test results for comprehensive judgement. The human–computer collaboration model facilitated by AI provides efficient and objective analysis and prediction, whereas doctors are responsible for the final clinical judgement and decision-making. This approach represents the cutting-edge use of AI-EEG to improve the precision and efficiency of epilepsy drug management.

#### 3.3.2 Surgical outcome assessment

Surgical outcome prediction similarly relies on Supportive AI technologies for the accurate identification and quantification of key biomarkers, such as HFOs. AI-EEG has significant value in surgical prognosis evaluation. For instance, high-sensitivity detection of HFOs can effectively improve postoperative outcome assessment in epilepsy surgery. This study used short-term energy (STE) and the Montreal Neurological Institute (MNI) detector to assess HFOs. By combining spike association and time-frequency plot characteristics, and employing DL techniques to purify pathological HFOs, it significantly improved the accuracy of predicting postoperative seizure outcomes (56). These findings indicate that AI has unique advantages in identifying key biomarkers. Automating the identification and purification of pathological HFOs remains a complex task, with varying performance among different algorithms. The universal predictive value of HFOs requires further validation across different centres and patient populations.

In addition, multimodal fusion technologies have also been explored for postoperative efficacy evaluation. For example, a study combined multimodal neuroimaging with video-EEG(v-EEG) to predict postoperative outcomes in patients with refractory epilepsy and explore prognostic predictors for these patients. This study collected data on demographics, clinical characteristics, v-EEG, neuroimaging, surgical details, and regular follow-up seizure outcomes. Multivariate analysis found that the multidisciplinary approach was an independent predictor of post-surgical outcomes in patients with intractable epilepsy (hazard ratio = 11.400, 95% confidence interval = 2.249–57.787, *p* = 0.003). This finding shows that the approach can provide independent prognostic information for patients with surgically refractory epilepsy. The result showed a statistically significant association. However, the very wide confidence interval (2.249–57.787) suggests considerable uncertainty in the estimate, which needs to be validated in studies with larger samples (57).

Furthermore, deep brain stimulation (DBS) is a neuromodulation technique that delivers adjustable electrical impulses to targeted cerebral nuclei, thereby offering a reversible, titratable, and non-lesional therapeutic strategy for patients with drug-resistant epilepsy (58).

Overall, AI-EEG technology, as a predictive AI tool, is gradually enhancing the ability to predict epilepsy treatment outcomes. Its effectiveness relies on a strong foundation of supportive AI, which is responsible for efficiently and accurately processing raw EEG signals, extracting information-rich features, and integrating multimodal data to provide reliable input for the final predictive modelling. However, the formulation of optimal postoperative management and follow-up strategies still requires clinicians to integrate the quantitative predictive information provided by AI, comprehensive preoperative assessments, specific surgical details, and individual patient factors for comprehensive judgement and decision-making. This human–computer collaboration model, where “AI provides in-depth analysis and where doctors lead clinical decision-making,” is an effective approach for optimizing epilepsy surgical treatment outcomes.

## 4 Challenges and future perspectives

### 4.1 Data limitations and generalization challenges

The efficacy of AI-EEG is highly dependent on high-quality training data. However, epilepsy research frequently encounters issues of data scarcity, limited sample sizes, and class imbalance. These constraints not only compromising the training performance of DL models but may also introduce classification bias toward majority classes, ultimately reducing the precision of AI-EEG in epilepsy diagnosis and treatment. For instance, one study analyzed extended EEG data (90-min recordings) from nine post-operative seizure-free patients, yet failed to encompass complete EEG monitoring. Incorporating more patients and expanding the EEG data scope could significantly improve the performance and reliability of DL algorithms (59).

Furthermore, many AI-EEG studies develop models using specific datasets, which can limit their performance when applied to different data populations. For example, one study used only EEG data from non-rapid eye movement (NREM) sleep stages for HFO detection. However, EEG recordings during wakefulness are influenced by multiple confounding factors—including subject movement, age, and vigilance states—which were not evaluated in this experimental design, ultimately compromising the model’s generalizability (56). Similarly, SCORE-AI has inherent constraints as it was exclusively developed and validated using routine EEG data, omitting neonatal and critically ill patient populations. Although routine EEG remains one of the most widely used recording modalities in clinical practice, this demographic limitation can significantly limit its applicability to specialized patient cohorts (11).

Another factor affecting generalization ability is inter-individual patient variability and dynamic changes in EEG signals. Electrical brain activity in different patients or in the same patient at different times dynamically changes with factors such as age and disease progression, rendering it challenging for generalized models to adapt to all individuals. For example, data from a study derived from pediatric patients at the same institution showed that as the age range expands and epileptic pathologies diversify, the morphology of HFOs may also change, thereby affecting the adaptability of algorithms (59). Additionally, research has indicated that long-term EEG monitoring data recorded during hospitalization may differ from EEG signals in patients’ daily living environments, potentially impacting the detection performance of wearable EEG devices (60).

### 4.2 Signal noise and interference issues

Although AI-EEG can reduce interference to some extent and improve detection accuracy, EEG signals remain susceptible to environmental noise such as power line interference and physiological artifacts like ocular movements and EMG, which can reduce detection accuracy. Studies have shown that approximately 21% of epileptic seizures are difficult to detect via bte-EEG, with main causes including unclear ictal EEG patterns, too short seizure duration, and seizures confined to the temporal lobe or limited by artifacts from postauricular electrode interference (61). Experimental studies have indicated that multi-device signals acquired from the left wrist, right wrist, or ankle may exhibit variations in signal quality and characteristics due to distinct physiological properties across different body regions, potentially compromising signal consistency (60). Seizures of unknown origin “are often accompanied by motor activities and noise artifacts, which can significantly interfere with EEG signal stability and complicate localization efforts (61).

In addition to environmental noise and physiological artifacts, the parameter limitations of signal acquisition itself, especially insufficient sampling frequency, is another important factor affecting the performance of AI-EEG. For example, studies have shown that a high sampling frequency with at least a sampling rate (FS) of ⩾2 kHz and an anti-aliasing filter (AAF) of ⩾500 Hz is a prerequisite for accurately capturing and identifying HFOs, which are important biomarkers of epileptogenic foci (62). However, many portable and wearable EEG devices are limited by power consumption, storage, and cost, with sampling frequencies typically in the range of 128–256 Hz. At this sampling rate, HFO signals cannot be fully captured, resulting in the loss of this valuable biological information. Even for the detection of low-frequency epileptiform discharges, a lower sampling rate may reduce the resolution of waveform details and increase the risk of misjudgment (23). Therefore, when evaluating the performance of AI-EEG systems, especially those involving HFO analysis or using portable devices, it is essential to fully consider whether the sampling frequency meets the frequency band requirements of the target signal.

### 4.3 Hardware limitations and computational resource bottlenecks

The practical application of many AI-EEG models is limited by insufficient hardware resources, leading to direct deployment challenges that impact model performance and generalization ability. For example, the limited number of SOZ-related independent components (ICs) in datasets presents challenges for traditional DL techniques in learning the complex features of these rare events (63). Additionally, due to computational resource constraints, a study set the frequency resolution at 2 Hz instead of 1 Hz. Lower frequency resolution can lead to loss of detail in epileptic seizure signals, thereby affecting detection accuracy (16).

Hardware limitations not only affect data processing capabilities but also restrict the selection of DL models. Due to Graphics Processing Unit (GPU) memory constraints, a study was limited in selecting CNN models and unable to incorporate more advanced network architectures such as Efficient-Net CNN and ResNet-50 CNN—models that demonstrate excellent performance in computer vision tasks. If applied to AI-EEG analysis, these models could provide richer feature extraction capabilities, thereby enhancing the accuracy of epileptic seizure detection and prediction (16). However, the potential value of these advanced models has been underutilized and unvalidated due to insufficient hardware resources.

Hardware limitations not only affect the complexity and selection of models but also directly restrict the quality of signal acquisition. High sampling frequencies, such as FS⩾2 kHz for HFO detection, exponentially increase the volume of data. This, in turn, places higher demands on the device’s storage capacity, wireless transmission bandwidth, as well as the computational processing power and storage space of the backend server (62). This further exacerbates the difficulty of deploying high-performance AI-EEG systems in resource-limited environments, such as wearable devices and primary healthcare institutions. Balancing the requirements of sampling rates with hardware feasibility is a significant challenge in current technological development.

### 4.4 Model explainability and clinical trustworthiness

The DL models that currently dominate the field of EEG analysis typically exhibit “black-box” characteristics, which conflict with the high demand for transparency in clinical decision-making. Although DL algorithms have shown high sensitivity and specificity in numerous experiments, their discriminative logic often deviates from neurophysiological interpretations, leading to limited trust in their outcomes among clinicians. This issue not only can hinder the clinical application of the model but may also reduce physicians’ acceptance of AI-assisted diagnostics. For example, the study by Ansari AH et al. showed that compared with heuristic algorithms that have clear decision paths and traditional MLmodels with strong interpretability, such as decision trees and rule-based systems, the decision-making process of complex DL models is relatively opaque (64). It is difficult to clearly explain why a particular EEG segment is classified as a seizure or non-seizure. Unlike traditional methods based on manually designed features and interpretable models, the features extracted by DL models are often mathematical representations obtained from EEG signals after filtering, pooling, and rectification, lacking a direct connection with neurophysiological mechanisms. This makes DL-based AI-EEG models difficult to provide clinically intuitive diagnostic evidence for physicians (19).

### 4.5 Future perspectives

Although significant progress has been made in epilepsy detection, monitoring, and treatment prediction using AI-EEG, future advancements will require overcoming multiple challenges and exploring new pathways. First, algorithm optimization is critical for enhancing AI-EEG performance. Optimizing DL models, particularly through techniques such as pruning, quantization, and distillation, can improve for improving computational efficiency, predictive accuracy, and reducing hardware resource demands (65). Additionally, adaptive training can enhance both supervised and self-supervised learning in deep neural networks, addressing issues such as data scarcity and sample imbalance, thereby improving model applicability and generalizability across diverse clinical settings. In the future, these approaches are anticipated to be integrated into EEG interpretation (66). Data augmentation and aggregation techniques, such as synthetic EEG data generation, time-window segmentation, and signal perturbation, have been suggested to enhance data diversity and optimize model learning capabilities (32). Furthermore, improving model architecture is a key direction for enhancing generalizability. For example, integrating multi-channel components, such as CNN-BLSTM, into frameworks like SZTrack can strengthen cross-channel dependencies and improve prediction accuracy (32).

Second, to address the issue of transparency and explainability, the integrating of explainable AI (XAI) can help simplify model complexity and increase clinicians’ trust in AI model judgments. For example, one study showed that significantly reducing the number of attributes and channels, and strategically selecting electrodes for model training, can contribute to the development of more effective mobile epilepsy detection applications (67). Future research should focus on incorporating more clinical expertise into AI-EEG models. Further research should be dedicated to embedding more clinical expertise into AI-EEG models, leveraging the automatic recognition of biomarkers and XAI technologies to ensure that AI can provide easily understandable decision support (60, 61). For instance, some researchers have suggested that models can be trained to recognize visual biomarkers used by human experts in EEG interpretation. However, this approach still needs further optimization. Recent studies have pointed out that the social barriers (data bias, patient safety, regulatory compliance) faced in the field of epilepsy prediction essentially stem from the untraceability of model decisions. However, this does not mean that researchers should revert to traditional transparent models, as such models may limit the upper bound of predictive performance. On the contrary, by infusing high-performance black-box systems with *post hoc* interpretability (such as feature attribution and decision path visualization), it is possible to unleash the technical potential of DL while meeting the needs of clinical trust (68).

Additionally, DL-based multimodal fusion models can enhance the matching between different modalities and strengthen clinical validation, thereby promoting clinical applications. Research has shown that the combination of EEG and fMRI has become a powerful tool for exploring brain function, and their synchronous measurement techniques are helpful for the development of clinical interventions (69, 70). Additionally, multidisciplinary collaboration, such as in neuroimaging, EEG, electronic health records, medical devices, and multimodal data integration, will be key to translating AI-EEG from experimental research to clinical practice (39). The combination functional and structural brain networks is more effective than using single-modality data alone. The joint construction of multimodal brain networks can further improve the matching and working ability of different data types (such as EEG, fMRI, PET/CT), increase the accuracy of models, and help promote the realization of personalized epilepsy treatment (71). Wearable devices and remote monitoring are important future directions. There is an urgent need to develop a new generation of low-power, high-sampling-rate, high-signal-quality, and user-friendly EEG sensing technologies. At the same time, research should be conducted on efficient edge computing algorithms to perform initial signal preprocessing and feature extraction on the device side, thereby alleviating the burden of high-sampling-rate data on transmission and cloud computing.

For clinical validation, future research should perform large-scale, multicenter, randomized controlled trials to validate the generalizability and effectiveness of AI-EEG across diverse patient populations. Standardization and regularization efforts are essential, including the establishment of unified evaluation criteria and technical specifications to ensure compatibility across different platforms and devices (19, 72, 73).

This includes establishing minimum technical specifications for EEG signal acquisition tailored to different application scenarios, such as routine monitoring, HFO detection, and seizure prediction, particularly clarifying the requirements for key parameters such as sampling frequency, number and placement of electrodes, common-mode rejection ratio (CMRR), and input noise. For research and applications aimed at capturing high-frequency biomarkers (such as HFOs), a sufficient sampling rate must be mandatorily required. Promoting device manufacturers to follow these specifications is crucial for ensuring the quality of signals input for AI-EEG analysis (74).

Finally, as AI-EEG technology becomes more widely applied, data privacy and security are as non-negligible challenges. To address this, researchers should implement data encryption techniques, blockchain, and other technologies to safeguard patient privacy and data security. Meanwhile, establishing regulatory frameworks is essential to ensure the fairness and accessibility of the technology. In the future, AI-EEG will need to address challenges such as data privacy and security, clinical validation and liability definition, technical fairness and accessibility. Balancing legal compliance, ethical considerations, and technical feasibility is the core task in advancing the practical application of AI-EEG (75, 76).

## Data Availability

The original contributions presented in the study are included in the article/supplementary material, further inquiries can be directed to the corresponding author.
